# A Redundant Tricuspid Valve Leaflet in a 23-Year-Old Female: A Report of a Rare Case and a Literature Review

**DOI:** 10.7759/cureus.43907

**Published:** 2023-08-22

**Authors:** Vikas Yadav, Roopeessh Vempati, Gurbina Sagoo, Ibrahim Youssef, Nenad Serafimovski

**Affiliations:** 1 Internal Medicine, Pandit Bhagwat Dayal Sharma Post Graduate Institute of Medical Sciences, Rohtak, IND; 2 Cardiology, Heart and Vascular Institute, Detroit, USA; 3 Internal Medicine, Gandhi Medical College & Hospital, Hyderabad, IND; 4 Internal Medicine, Maharshi Markandeshwar Institute of Medical Sciences and Research, Ambala, IND; 5 Internal Medicine, Valley Health System, Las Vegas, USA

**Keywords:** valvular mass, pulmonary artery hypertension, tricuspid regurgitation, redundant tricuspid valve, accessory tricuspid valve

## Abstract

Accessory tricuspid valve (ATV) is a rare cardiac anomaly often associated with other complex cardiac disorders, most commonly tetralogy of Fallot (TOF) and ventricular septal defect (VSD), but it can also be an isolated entity. It is usually diagnosed in childhood. It can be asymptomatic and get diagnosed incidentally, but it can also lead to cardiac complications such as right ventricular outflow obstruction (RVOTO) or tricuspid valve regurgitation.

Here, we present the case of a 23-year-old woman with an isolated ATV diagnosed on an echocardiogram without symptoms after a physical exam noted a murmur, with a subsequent echocardiogram showing moderate to severe tricuspid valve regurgitation.

A literature review was also performed. Only a small number of ATV cases have been reported, most of them diagnosed in childhood and associated with other congenital cardiac anomalies. Most of the cases necessitated surgical intervention, with a portion resulting in fatalities. It is important to understand the diagnosis and morphology of the ATV due to its association with dangerous complications to guide the management of this entity. It is also important to conduct further research to understand the inheritance of this pathology and develop screening and prevention plans.

## Introduction

The accessory tricuspid valve (ATV) leaflet is a rare anatomical finding that is often observed in children with complex congenital heart defects [1]. This anatomical anomaly can be either fixed or mobile. The fixed anomaly is usually found attached to the interventricular septum with short chordae tendineae, while the mobile leaflet can be found floating freely in the right ventricle or in the right ventricular outflow tract, causing obstruction [1].

This anomaly may look like cardiac vegetation on transthoracic echocardiography and can lead to the misdiagnosis of infective endocarditis, especially in patients with a clinical profile suggesting an infection [2]. In addition, this is usually associated with a ventricular septal defect (VSD), where the redundant leaflet can occlude the defect partially or completely [1].

The diagnosis of this rare anomaly is usually incidental while performing echocardiography, cardiac computerized tomography (CT), or cardiac magnetic resonance imaging (MRI) [1]. A definitive diagnosis is made by surgery, where intraoperative findings may show a myxomatous redundant leaflet that displays a thickened and fibrosed spongiosa layer in histopathological examination [1, 2].

An ATV leaflet is infrequently reported in the medical literature. Here we present the case of a patient with this pathology.

## Case presentation

At the age of five, a 23-year-old woman without a significant past medical history was found to have a murmur during her visit to a pediatrician for an unrelated minor ailment. An echocardiogram was conducted to further evaluate the murmur, revealing an accessory tricuspid valve leaflet.

The patient remained without cardiac-related symptoms until she was 17 years old, at which point she experienced chest pain and palpitations for the first time. She sought care from pediatric cardiology, and an echocardiogram performed at that time showed similar findings as a prior study conducted 12 years earlier.

Upon reaching the age of 21, the patient transitioned her care to adult cardiology at our institution. A recent echocardiogram, conducted last year, revealed that the tricuspid valve leaflet had accessory tissue, leading to redundancy (Video 1).

**Video 1:** A 2D echocardiogram in apical four chamber view showing accessory tricuspid valve leaflet

**Figure 1:**
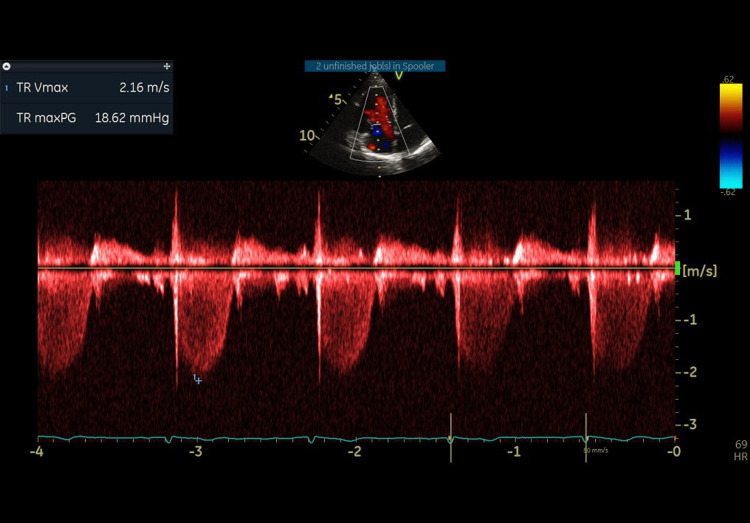
A continuous wave-Doppler across the tricuspid valve in severe tricuspid regurgitation

This was associated with moderate to severe tricuspid regurgitation (Videos 2-3, Figure 1).

**Video 2:** A 2D Doppler echocardiogram in severe tricuspid regurgitation taken in the right ventricular inflow view

**Video 3:** A 2D Doppler echocardiogram in severe tricuspid regurgitation taken in the right ventricular-focused apical four chamber view

The patient recently underwent a treadmill stress test that was negative and an event monitor study for seven days that showed sinus tachycardia. She has not been diagnosed with any other cardiac disorders. At present, she is recommended for regular follow-ups, and we will closely watch for symptoms and signs of congestive heart failure.

## Discussion

We conducted a comprehensive literature search using PubMed, Google Scholar, and Scopus databases. The search was performed using the keywords "accessory tricuspid valve," "extra tricuspid valve," "redundant tricuspid valve," "accessory tricuspid valve tissue," "extra tricuspid valve tissue," and "redundant tricuspid valve tissue." The search was limited to titles and abstracts (TIAB) to ensure a broad search and capture relevant articles. Boolean operators (OR) were used to combine the different variations of the search terms.

After the initial search, articles were screened based on their titles and abstracts. We included articles that reported cases, case series, or articles specifically discussing the accessory tricuspid valve or its related terms. The articles obtained were manually reviewed. Full-text accessibility was considered a criterion for inclusion. A total of 31 cases matching our criteria were found, including 17 full-text articles and two abstracts without full-text availability. Six articles were excluded due to inaccessibility.

Data from the included articles was extracted using a standardized data extraction form. We collected information on author names, publication year, study design, patient demographics, clinical presentation, diagnostic methods, any family associations, and treatment strategies (Table 1).

**Table 1:** Cases found on Literature review RVOTO - Right ventricular outflow tract obstruction; TTE- Transthoracic echocardiogram; TEE - Transesopahgeal echocardiogram; VSD - Ventricular septal defect; TOF - Tetrology of Fallot; SOB - Shortness of breath; TV - Tricuspid valve

Sr.	Author	Published Year	Age (in Years)/ Gender	Presentation	Associated Condition	Family History	Diagnostic Modality	Treatment	Note
1	Andrew Yoon et al [1]	2008	60/F	Asymptomatic	N/A	N/A	echocardiogram, Cardiac MRI and Biopsy	Surgical removal	The asymptomatic case
2	Nishith Bhargava et al [2]	2015	17/M	Cyanosis	RVOTO	N/A	TTE, TEE	Not removed, just repaired	Presented as an infective endocarditis
3	Michael A. LaCorte et al [3]	1985	4/M	Cyanosis	TOF	N/A	TTE	Not removed, just repaired	N/A
4	Chew Lee et al [4]	2010	29/M	SOB	RVOTO	N/A	TTE	Surgical removal	Isolated accessory TV producing mild RVOTO
5	Prabhat Tewari et al [5]	2006	5/M	SOB	TOF	N/A	TTE	Surgical removal	N/A
6	Faggian et al [6]	1983	N/A	N/A	TOF	N/A	N/A	N/A	case series of four cases, all 4 associated with TOF
7	Neufeld et al [7]	1960	N/A	N/A	TOF	N/A	N/A	Surgical removal	A series of three cases (2 males and 1 female), all associated with TOF. 1 died before surgery, and 2 had surgical removal.
8	Yoshimura et al [8]	2000	N/A	N/A	TOF	N/A	N/A	Not removed, just repaired	A case series of 8 cases, 6 had TOF, 2 had other cardiac co-morbidities, all repaired surgically
9	Pate et al [9]	1968	19/M	SOB, Cyanosis	RVOTO	N/A	Cineradiography	Surgical removal	N/A
10	Ehrenhaft et al [10]	1959	4.5/M	Murmur	RVOTO	N/A	Intra-operative	Surgical removal	Looked like VSD + Pulmonic Stenosis
11	Gajjar et al [11]	2018	0.7/M	failure to thrive	VSD	N/A	TEE	Surgical removal	N/A
12	Negi et al [12]	2020	3/M	Cyanosis	VSD	N/A	TEE	Surgical removal	N/A
13	Ozturk et al [13]	2018	41/M	Atypical Chest pain	N/A	N/A	TEE	N/A	N/A
14	Mahipat et al [14]	2012	12/M	SOB, Cyanosis	VSD	L Hemiparesis	TTE	Not removed, just repaired	N/A
15	Nabati M et al [15]	2014	17/M	SOB, Cyanosis	Congenital HD	N/A	TTE	N/A	Surgery denied by the patient
16	Shetkar SS et al [16]	2015	10/M	Cyanosis	RVOTO	N/A	TTE	N/A	N/A
17	Krest G et al [17]	2010	newborn/M	Cyanosis	RVOTO	N/A	TTE	Surgical removal	N/A
18	Miche E et al [18]	1991	27/M	SOB	RVOTO	N/A	TTE	Surgical removal	N/A
19	Z G Mesko et al [19]	1978	3.7/F	N/A	VSD	N/A	Intraoperative	N/A	N/A

The presence of ATV leaflets is a rare finding, particularly in individuals without complex congenital cardiac malformations. It is more commonly reported in children with associated congenital defects such as tetralogy of Fallot (TOF), transposition of the great arteries, and VSDs [1, 2]. In contrast, accessory mitral valve leaflets are typically attached at the junction of the aortic valve and anterior mitral leaflet and are associated with complex congenital cardiac malformations [1].

While there are numerous reports on ATV tissue, there are a limited number of cases where accessory mitral and tricuspid valve leaflets occur in the same patient [1]. The diagnosis of the ATV leaflet can be suspected through non-invasive imaging modalities such as echocardiography, cardiac CT, or cardiac MRI [1].

The clinical presentation of patients with ATV leaflets can vary. Some individuals may remain asymptomatic, especially when the accessory leaflet is not accompanied by other complex cardiac malformations [1]. Asymptomatic adults with atypical valvular lesions should also be considered for the diagnosis of ATV leaflets [1].

Accessory tricuspid valve leaflets can cause obstructions in various ways. In cases where VSDs are present, both the fixed and mobile types of ATV can partially or completely obstruct the VSD [2, 3, 4]. The mobile-type ATV leaflet can even migrate through the VSD and cause right ventricular outflow tract obstruction (RVOTO) [2].

There have been reports of ATV leaflets associated with anomalies of the tricuspid valve, such as up to six cusps and other valvular malformations [5]. Some studies have also described sudden death in association with cusp and chordae tendineae malformations of the tricuspid valve [5]. Therefore, careful evaluation of the tricuspid valve is important in these cases.

Two-dimensional transthoracic echocardiography, particularly subcostal long-axis, and short-axis views, as well as right ventricular angiography, are useful diagnostic methods for identifying ATV leaflets [5]. However, it should be noted that ATV tissue can be histopathologically similar to normal valve tissue, making diagnosis challenging [5].

The presence of ATV leaflets is significant from both clinical and hemodynamic perspectives, as it can mimic severe pulmonary stenosis or atresia with an intact interventricular septum [6]. The morphology of the ATV tissue is crucial in determining the appropriate surgical approach. Mobile-type accessory leaflets must be resected during surgical repair, while fixed-type leaflets can be utilized for suture anchorage during VSD closure [6].

In cases of ATV with small VSDs, the clinical presentation can resemble severe pulmonary stenosis with either an intact ventricular septum or a small VSD and a right-to-left shunt at the ventricular level [7].

Histological analysis of the ATV has revealed normal structure in the fixed type, while the mobile type has shown thickening of the spongiosa layer, potentially due to chronic mechanical load [8]. This suggests that the thickening of the accessory valve tissue may be an acquired lesion [8]. A third type of ATV, combining features of both the mobile and fixed types, has been identified [11]. This type, depending on the attachment of the chordae, can lead to either RVOTO or right ventricular inflow tract obstruction [11]. If the ATV tissue is not excised during VSD repair surgery, it may cause RVOTO [12].

## Conclusions

In conclusion, ATV leaflets are rare findings, often associated with complex congenital cardiac malformations, most likely TOF and VSD. They can cause obstruction in various ways, partially or completely occluding ventricular septal defects and potentially leading to RVOTO. Tricuspid valve leaflets not associated with other congenital cardiac malformations may not be diagnosed until late childhood or adulthood. Although they can be incidental findings, they may be associated with severe regurgitation and close follow-up is needed to avoid complications. Proper diagnosis and understanding of the morphology of this pathology are essential, as surgical management may be necessary. Further research is needed to fully understand the inheritance of this disorder for potential screening recommendations and to fully recognize the clinical implications and long-term outcomes associated with this condition.
