# Correction: A Redundant Tricuspid Valve Leaflet in a 23-Year-Old Female: A Report of a Rare Case and a Literature Review

**DOI:** 10.7759/cureus.c143

**Published:** 2023-11-16

**Authors:** Vikas Yadav, Roopeessh Vempati, Gurbina Sagoo, Ibrahim Youssef, Nenad Serafimovski

**Affiliations:** 1 Internal Medicine, Pandit Bhagwat Dayal Sharma Post Graduate Institute of Medical Sciences, Rohtak, IND; 2 Cardiology, Heart and Vascular Institute, Detroit, USA; 3 Internal Medicine, Gandhi Medical College & Hospital, Hyderabad, IND; 4 Internal Medicine, Maharshi Markandeshwar Institute of Medical Sciences and Research, Ambala, IND; 5 Internal Medicine, Valley Health System, Las Vegas, USA

This article has been corrected to include a missing table and text that was accidentally deleted during the editing process. The journal greatly regrets that this error occurred and was not identified prior to publication. The erroneously removed section of the article consisted of the first three paragraphs of the Discussion section along with Table 1. This content has now been restored and can be found in the body of the article. Once again, the journal deeply regrets this error.

